# Luteal phase stimulation versus follicular phase stimulation in poor ovarian responders: results of a randomized controlled trial

**DOI:** 10.1186/s12958-020-00570-7

**Published:** 2020-02-07

**Authors:** Joaquín Llácer, Belén Moliner, Lydia Luque, Andrea Bernabéu, Belén Lledó, Juan Carlos Castillo, Jaime Guerrero, Jorge Ten, Rafael Bernabéu

**Affiliations:** 1 Department of Reproductive Medicine, Instituto Bernabeu, Av. Albufereta 31-37, 03016 Alicante, Spain; 2 Department of Clinical Laboratory, Instituto Bernabeu, 03016 Alicante, Spain

**Keywords:** Follicular-phase ovarian stimulation, Luteal-phase ovarian stimulation, Randomized clinical trial, Ovarian reserve, Poor ovarian responders

## Abstract

**Background:**

In young women with poor ovarian response, luteal-phase ovarian stimulation (LPOS) is a potential method for collecting competent oocytes. The aim of this study was to assess the efficacy of LPOS compared with follicular phase ovarian stimulation (FPOS) in young women with poor ovarian response (POR).

**Methods:**

This single-center, prospective, randomized pilot study compared LPOS and FPOS in women with POR fulfilling Bologna criteria who underwent in vitro fertilization at the Instituto Bernabeu. The primary outcome was the number of metaphase II (MII) oocytes obtained by follicular puncture.

**Results:**

Sixty women were included in the study, with 27 women completing LPOS and 30 undergoing FPOS. There was no statistically significant difference in the number of MII oocytes obtained between the LPOS group and the FPOS group (2.1 ± 2.0 vs. 2.6 ± 2.2, *p* = 0.31). Length of stimulation was also similar in both groups (8.35 ± 2.8 vs. 8.15 ± 4.1 days, *p* = 0.69). Similarly, there was no significant difference in the follicle-stimulating hormone total dose, number of cumulus-oocyte complexes, survival rate, fertilization rate, or cancellation rate between groups. A significantly higher Ovarian Sensitivity Index was observed in the LPOS group versus the FPOS group (0.96 vs. 0.57, *p* = 0.037).

**Conclusion:**

LPOS was comparable with FPOS in terms of efficacy and may improve ovarian responsiveness in young women with POR.

**Trial registration:**

ClinicalTrials.gov identifier: NCT02625532; EudraCT identifier: 2015–003856-31.

## Introduction

Poor response to controlled ovarian stimulation is one of the greatest challenges in assisted reproduction technology and has been reported to occur in 9–24% of women undergoing in vitro fertilization (IVF) [1]. Data from the Society for Assisted Reproductive Technology (SART) and the American Society for Reproductive Medicine (ASRM) registry showed that at least 50% of cancelled cycles are in women with poor response [2].

The European Society for Human Reproduction and Embryology (ESHRE) defined poor ovarian response (POR) in IVF according to the presence of at least two of the following three features: (1) advanced maternal age or any other risk factor for POR; (2) a previous POR; and (3) an abnormal ovarian reserve test [3]. Based on this definition, POR has been reported to occur in 10.3% of cases [4].

Although many stimulation protocols have been established to improve clinical outcomes in women with POR, the protocol that is the most effective remains controversial and there is inadequate evidence to recommend any ovarian stimulation protocol as more effective in this population [5, 6].

Initiating ovarian stimulation in the early follicular phase is essential for fresh transfer and for the endometrium to be receptive during that cycle. However, due to advances in the field of cryobiology [7, 8], this is no longer necessary. Ovarian stimulation can conclude with elective freezing of oocytes or embryos, with similar results to those of a fresh embryo transfer [9] even in patients with poor response [10]. In fact, one of the proposed strategies in patients with poor response is the accumulation of oocytes for subsequent fertilization [11].

Previous studies demonstrated the appearance of more than one wave of follicular growth within a cycle, suggesting the presence of obtainable follicles during the luteal phase [12]. Although ovarian stimulation during the luteal phase was reserved for women with cancer in whom oncological treatment could not be delayed [13–15], in recent years, luteal phase stimulation has been identified as an adequate method of obtaining a sufficient number of competent oocytes [16]. This offers the possibility of collecting oocytes twice in the same cycle in order to obtain the highest number of eggs in the shortest period of time [17–19]. Results of double stimulation in poor responders suggest a better response in the second stimulation during the luteal phase, but this effect could be explained by priming of stimulation in the follicular phase [17]. Thus, the efficacy of ovarian stimulation in the luteal phase of women with POR compared with conventional protocols is yet to be determined [20–22].

The objective of this prospective pilot study was to assess whether luteal-phase ovarian stimulation (LPOS) presents similar efficacy in terms of oocyte yield compared with stimulation in the conventional follicular phase in young women with POR.

## Methods

### Study design

This single-center, prospective, randomized pilot study (ClinicalTrials.gov identifier: NCT02625532; EudraCT identifier: 2015–003856-31) assessed the efficacy of follicular-phase ovarian stimulation (FPOS) compared with LPOS in women with POR fulfilling Bologna criteria.

The study was approved by the Ethical Committee of San Juan (Alicante, Spain). Written informed consent was obtained from all participants.

### Study participants

Data were collected from women with POR who underwent IVF treatment at the Instituto Bernabeu (Alicante, Spain) between February 2016 and December 2017. Inclusion criteria were: poor responders (Bologna Criteria) [3], age < 41 years, regular menstrual cycles of 21–35 days, indication for IVF, indication for starting stimulation with 300 IU of follicle-stimulating hormone (FSH), presence of both ovaries, ability to participate and comply with the study protocol and having signed the written consent form. Women with follicles > 10 mm in the randomization visit, endometriosis stage III/IV, concurrent uterine pathology (e.g. adenomyosis, submucosal myomas, Asherman’s syndrome) and concurrent participation in another study were excluded. All patients had at least one previous cycle with less than 4 oocytes and altered ovarian reserve parameters.

### Interventions

Women were randomized into two groups: the study group that initiated ovarian stimulation in the luteal phase (LPOS group) and the control group that initiated ovarian stimulation in the follicular phase (FPOS group).

Patients in the LPOS group performed daily urinary luteinizing hormone (LH) tests from day 7 of their cycle and started administration of two vials of 150 IU recombinant FSH plus 75 IU recombinant LH (Pergoveris® 150/75) daily from the fourth day of the positive LH test. Patients in the control FPOS group started ovarian stimulation at day 2 or 3 of the cycle with two vials of 150 IU recombinant FSH plus 75 IU recombinant LH (Pergoveris® 150/75). In both groups, administration of the GnRH antagonist cetrorelix acetate (Cetrotide®) was started when the largest follicle was ≥14 mm, thereafter, examination was performed every 24–72 h with ultrasound assessment and blood hormone analysis with determination of estradiol and progesterone levels and 2 vials of 0.1 mg triptorelin acetate (Decapeptyl®) were administered when at least one follicle reached ≥18 mm in diameter.

### Oocyte retrieval and fertilization

Oocyte collection was performed by transvaginal ultrasound-guided puncture 36 h after administration of triptorelin according to the protocol of our institution. The Kitazato method using the Cryotop device was used for oocyte vitrification/warming as described elsewhere [23].

Vitrification was carried out 2 h after oocyte retrieval and immediately after nuclear maturity assessment. Warmed oocytes were cultured for 2 h prior to intracytoplasmic sperm injection (ICSI). The standard dosages and protocols were used for patients diagnosed with POR. Successful fertilization was defined as two clear pronuclei being present 16–18 h after insemination.

### Randomization

Randomization was performed between day 2–3 of the menstrual cycle according to a list of random allocation of treatments. After checking that there were no contraindications to start the stimulation, the patients were assigned to the treatment group. The randomization list was generated by the statistical program SAS® (PLAN procedure, Copyright (c) 2002–2012 by SAS Institute Inc., Cary, NC, USA), in such a way that both treatments have an equal probability of being assigned. Investigators had no access to this list. Treatment allocation was placed in a sealed, opaque envelope and picked up consecutively by a nurse at the moment of randomization. Patients were included in the study consecutively from the inclusion of the first eligible patient according to the screening criteria. Randomized treatment was assigned immediately after the patient had confirmed inclusion in the study. The study was not blinded.

### Sample size

In total, a sample size of 60 patients (30 for each group) was estimated to be sufficient to analyze the efficacy of LPOS compared with FPOS based on previous studies [24, 25]. In the study of Kim and colleagues, 24 patients were required to be included in each group in order to detect a difference of 1.5 cumulus-oocyte complexes (COCs), using a two-sided, Mann–Whitney test with 80% power, given a standard deviation (SD) of 1.9 and a significance level of 0.05 [25]. A difference of 1.5 COCs retrieved, on which the power analysis was performed, is based on the results of a subsequent meta-analysis [24], which showed that testosterone pretreatment increased the number of COCs by 1.5. Secondly, the difference of 1.5 COCs would likely result in approximately one embryo difference between groups, assuming a fertilization rate of 65%. A difference of 1.5 COCs and thus of one embryo is likely to be crucial in a proportion of poor responders, since it may differentiate between those who will proceed to embryo transfer, and thus retain the possibility to achieve pregnancy, and those who will not proceed to embryo transfer.

### Outcomes

The primary outcome of this study was the number of oocytes in metaphase II (MII) obtained by follicular puncture. The length of stimulation, FSH total dose, number of COCs obtained by follicular puncture, survival rate (after thawing), and fertilization rate were considered as secondary outcomes. Fertilization rate was defined as the number of correctly fertilized oocytes 18 h post-insemination, and cancellation rate was defined as the ratio of cancelled cycles to the number of initiated ovarian stimulation cycles. A post-hoc analysis was carried out to assess the ovarian sensitivity index (OSI) in both groups. OSI was calculated by dividing the total number of COCs retrieved by the anti-Müllerian hormone (AMH) level.

### Statistical analysis

Categorical variables are presented as percentages with 95% confidence intervals. Continuous variables are presented as mean ± SD, and range. Statistical analysis was performed with SPSS version 22.0 software (SPSS, Chicago, IL, USA). In order to compare the data between the two groups, we used the student’s t-test for cuantitative variables. The categorical variables were analyzed with the χ^2^ test. A *p* value of < 0.05 was considered statistically significant.

## Results

### Study participants

**Fig. 1:**
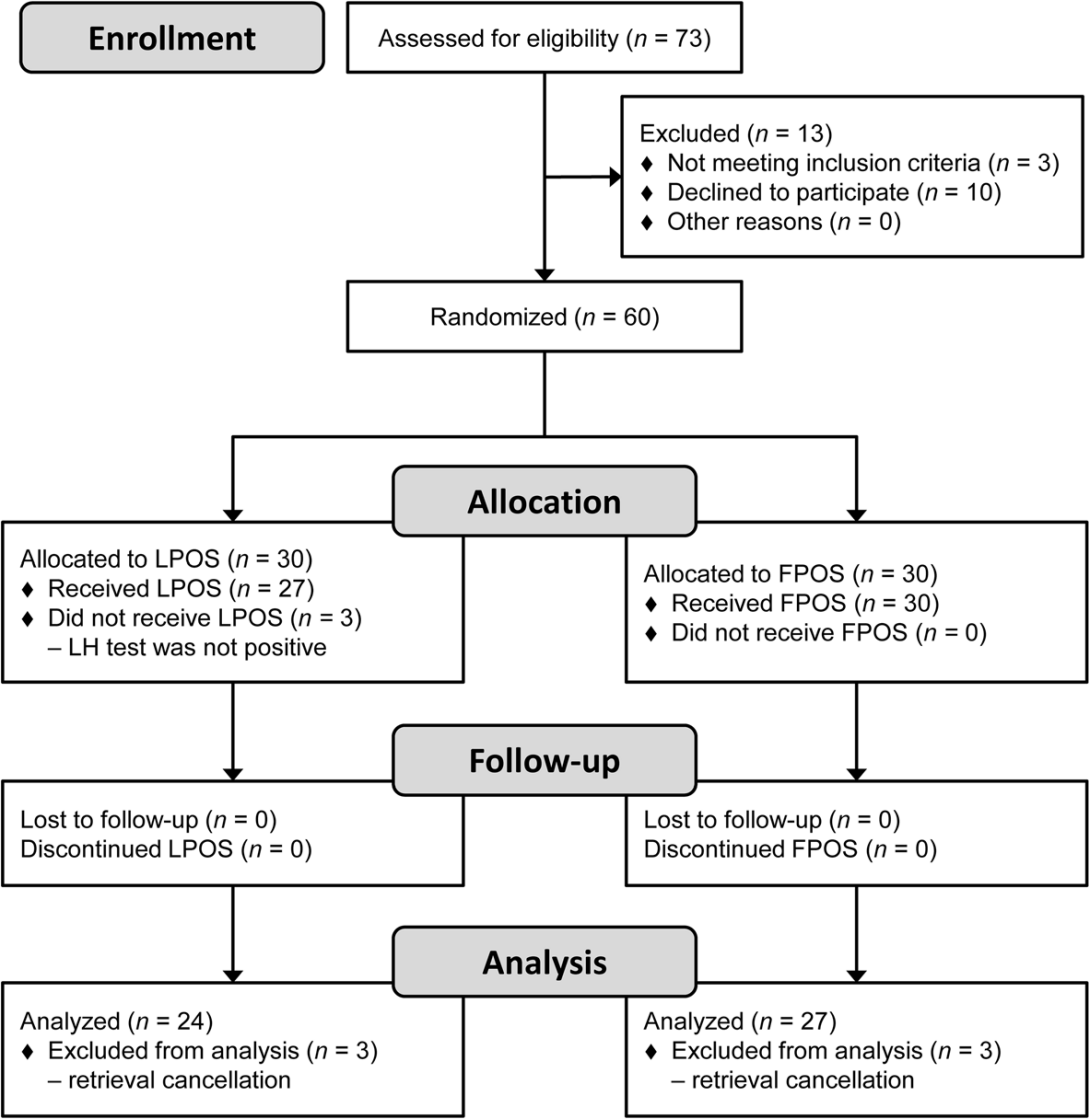
Flow-chart of the study

In total, 60 women with POR who underwent IVF treatment between February 2016 and December 2017 were included in the study. Figure 1 shows the participant flow in the study. After randomization, ovarian stimulation was completed for 27 patients with LPOS and 30 with FPOS.

### Baseline characteristics

Patients had a mean ± SD age of 36.1 ± 3.22 years in the LPOS group and 35.6 ± 2.62 years in the FPOS group. There were no significant differences in patients’ age, body mass index (BMI), weight, years of infertility, or basal antral follicle count (AFC) between both groups (Table 1). The basal AMH was significantly lower in the LPOS group than in the FPOS group.

**Table 1:** Baseline characteristics of poor ovarian responders in the follicular and luteal phase stimulation groups

	LPOS	FPOS	*p* value^a^
Age, years	36.1 ± 3.2	35.6 ± 2.6	NS
BMI, kg/m^2^	24.8 ± 0.7	23,7 ± 0.6	NS
Weight, kg	66.1 ± 11.4	63.1 ± 9.4	NS
Previous ovarian stimulations	2.1 ± 1.1	2.0 ± 1.1	NS
Previous mean eggs retrieved	2.0 ± 1.3	2.1 ± 1.2	NS
AFC, *n*	4.0 ± 3.2	4.7 ± 2.6	NS
AMH, pmol/L	4.7 ± 3.8	7.3 ± 4.8	0.021

Values are presented as mean ± standard deviation

*BMI* body mass index, *AFC* antral follicle count, *AMH* Anti-Müllerian hormone, *NS* not significant

^a^Calculated using student t-test

### Clinical outcomes

There was no statistically significant difference in the number of MII oocytes between the LPOS group and the FPOS group (Table 2). Similarly, no statistically significant difference in the number of COCs, the length of stimulation, total FSH dose, the cancellation rate, survival rate or fertilization rate was observed between both groups (Table 2).

**Table 2:** Comparison of clinical outcomes from LPOS and FPOS protocols

	LPOS	FPOS	*p* value	Mean Difference (95% CI)
Oocytes MII, mean ± SD	2.1 ± 2.0	2.6 ± 2.2	0.31^a^	0.57 (− 1.67, 1.14)
COCs, mean ± SD	2.7 ± 2.5	3.1 ± 2.1	0.44^a^	0.47 (− 1.66, 0.73)
Length of stimulation, mean ± SD, days	8.4 ± 2.8	8.2 ± 4.1	0.69^a^	0.2 (− 2.5, 2.1)
Total FSH dose, mean ± SD, IU	2505 ± 913	2445 ± 690	0.76^a^	65 (353.5, 483.5)
Cancellation rate, %	20	10	0.09^b^	1.6 (0.61, 4.15)
Survival rate, %	94.9	83.6	0.09 ^a^	1.01 (0.98, 1.04)
Fertilization rate, %	68.9	65.7	0.78^a^	1.01 (0.99, 1.04)
OSI	0.96	0.57	0.04	0.39 (0.02, 0.75)

*COCs* cumulus-oocyte complexes, *FSH* follicle-stimulating hormone, *OSI* Ovarian Sensitivity Index, *MII* metaphase II, *SD* standard deviation

^a^Calculated using student t-test

^b^Calculated using χ^2^ test

### Hormone levels in the LPOS group

**Fig. 2:**
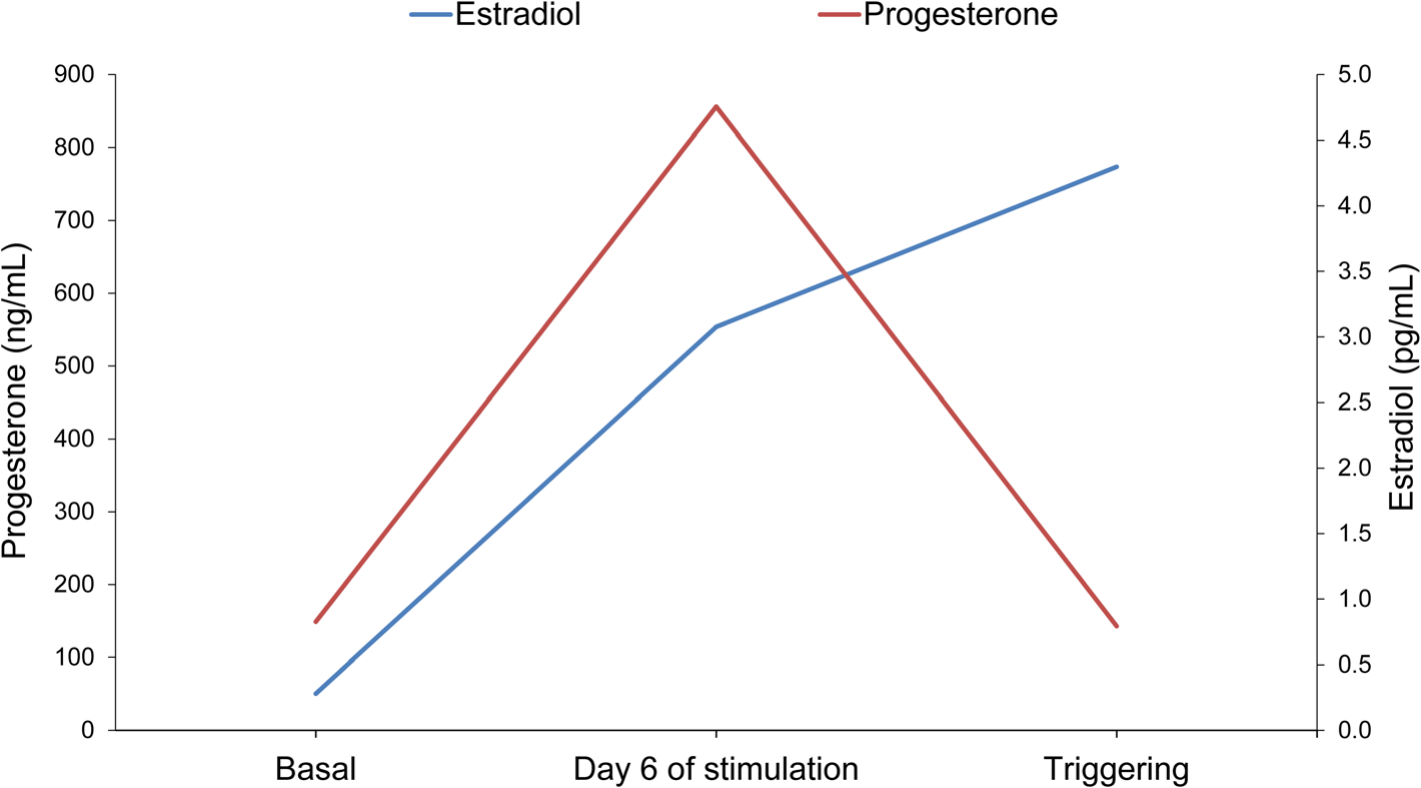
Hormone levels in the luteal phase ovarian stimulation (LPOS) group

Progesterone and estradiol levels were assessed in the LPOS group (Fig. 2). The progesterone levels at the moment of triggering were similar to the basal preovulatory levels.

### Post-hoc analysis of ovarian sensitivity index

Taking into account the higher AMH levels in the FPOS group, a post-hoc analysis showed a significantly greater OSI in the LPOS group compared with the FPOS group (0.96 vs. 0.57, *p* = 0.037).

## Discussion

To the best of our knowledge, this is the first randomized controlled study comparing the efficacy of FPOS and LPOS in a population of women with POR fulfilling Bologna criteria. These results show that LPOS has comparable efficacy to FPOS and suggest that it may improve ovarian response in young women with POR.

In our study, both the number of mature oocytes collected and the duration of treatment were similar in both groups, so for patients who accumulate oocytes or embryos, waiting for the onset of menstruation to initiate ovarian stimulation may no longer be necessary. Therefore, this can reduce the time needed to obtain the largest number of oocytes or embryos in the shortest time possible, which is of vital importance in patients diagnosed with POR and/or advanced age.

Previous studies of the efficacy of LPOS compared with FPOS in women with POR are scarce. A randomized, controlled study in 40 women with POR found that patients undergoing LPOS had similar numbers of oocytes retrieved compared with those undergoing FPOS [26]. A more recent randomized, open-label pilot trial of 18 women with POR confirmed that the number of oocytes retrieved is similar regardless of the stimulation phase [27]. In addition, this trial did not find significant differences between the two stimulation regimens with regard to other endpoints, such as follicular growth, serum estradiol levels, pregnancy, and live birth rates [27]. Another pilot study of a larger population of women with PORs (*n* = 60), similar in size to our study, showed that the numbers of retrieved oocytes, MII oocytes, fertilized oocytes and day-3 embryos were significantly higher in the LPOS group than in the FPOS group [28].

The main strength of our study is that exactly the same dose and stimulation protocol were used for FPOS and LPOS, avoiding potential bias due to the use of different protocols, such as those reported in the Shanghai protocol [18] or the previous pilot study [28], where despite their prospective and randomized designs, different doses and gonadotropins were used, as well as different strategies to inhibit the LH peak. One of the most important aspects to consider is that the duration of the stimulation was similar in both groups. The authors of the Shanghai protocol stated that the use of letrozole was necessary in the LPOS to avoid an excessive lengthening of stimulation [18], but our results challenge this hypothesis.

Another strength of our study is the assessment of LPOS separately from double ovarian stimulation, which avoids the possible priming effect of the previous stimulation during the follicular phase of the same cycle. A recently published case-control study in 188 poor prognosis patients with paired follicular phase- and luteal phase-derived cohorts of oocytes collected after stimulations in the same ovarian cycle (DuoStim) found that LPOS-derived oocytes are as competent as FPOS-derived oocytes [17]. This finding supports the use of LPOS for poor prognosis patients and questions the ‘single recruitment episode’ theory of follicle recruitment [17]. Previous studies have also reported a significantly higher number of oocytes collected after LPOS than after FPOS [16], which may have been influenced by the DuoStim approach itself, since LPOS is conducted soon after FPOS is ended. Therefore, the high levels of estradiol and progesterone reached after FPOS may synchronize the cohort of antral follicles that will grow during LPOS, as well as boost the proliferation of FSH receptors in their granulosa cells [29], resulting in an overall better response to ovarian stimulation.

Our study did not show a greater response to ovarian stimulation with LPOS versus FPOS. This situation could be explained by the study population included, since the patients who meet the Bologna criteria constitute a group with an especially poor prognosis [30]. Therefore, it is extremely difficult for any strategy to prove useful, and this classification has been the subject of criticism [31]. On the other hand, and despite the strict randomization process, patients assigned to the FPOS group had higher AMH levels and a greater response was expected in this group, which in fact did not occur. In our study, the ratio of the absolute number of oocytes retrieved and the AMH level (i.e. the OSI) was significantly higher in the LPOS group compared with the FPOS group. Some authors hypothesize that this ratio is a better representation of ovarian responsiveness than either parameter on its own [32]. The use of OSI instead of the number of retrieved oocytes as the measure of ovarian responsiveness seems to be more appropriate and particularly useful because different patients showed different AMH levels, which could have a confounding effect on the results. Based on this scenario, LPOS may be considered a better option to obtain a higher ovarian responsiveness. A randomized study with a larger sample size and a different population including suboptimal responders [33] is mandatory to definitively assess this possibility.

The results of the hormone assessments in the LPOS group were of special interest. First, the levels of estradiol had an ascending evolution in values comparable to that of the cycles with conventional stimulation, so this may be of interest for a better stimulation control. On the other hand, with regard to progesterone levels, a significant rise was observed after 6 days of stimulation, providing evidence of activity in the corpus luteum. However, progesterone levels on the day of the triggering were similar to the preovulatory ones. This fact points to the need for the use of strategies to inhibit the LH peak during LPOS, either with antagonists or with exogenous progesterone administration, since these levels do not ensure an effective blockade of estradiol-stimulated surges in LH [34, 35].

No differences were observed in the subsequent evolution of the oocytes obtained in both the LPOS and FPOS groups. The survival rate after thawing and the fertilization rate were similar in both groups, so it does not seem that the use of LPOS or FPOS influences oocyte quality. Due to the design of the study (patients who accumulated oocytes for later use together with other oocytes collected from different stimulations), clinical results could not be obtained. However, the results of previous studies are reassuring in this regard, showing similar rates of blastocyst formation, aneuploidy, pregnancy, live birth rates, obstetric outcomes and live birth defects [17, 36].

The implication of our study results is the possibility of elective vitrification of oocytes or embryos in young women with POR with LPOS using the same protocol as FPOS and without needing to wait the whole menstruation cycle. Moreover, LPOS may offer advantages over traditional protocols, such as shortened periods towards follicular stimulation and a higher yield of oocytes retrieved per started cycle [37], which could be particularly attractive in patients with an initial POR.

Finally, several limitations of our study should be considered. Firstly, our results are based on a single-centre, non-blinded, pilot trial with a small sample size that did not perform a sample size calculation before initiating enrolment. Secondly, the participants who were enrolled on the basis of Bologna criteria [3] might be heterogeneous. Lastly, no stratification of the sample considering different levels of AMH was performed. Thus, caution should be taken in generalizing this finding and larger multicentre randomized studies are mandatory to confirm the best option for ovarian stimulation in this population. More studies need to be conducted in the future to confirm the safety of LPOS, in terms of ovarian (and follicular) environment as well as clinical, peri-natal and post-natal outcomes.

## Conclusion

The results of our study suggest that LPOS has comparable efficacy to FPOS in terms of number of MII oocytes retrieved and may improve ovarian responsiveness in young women with POR. Future randomized controlled trials with a larger sample size are encouraged to elucidate the best strategy of ovarian stimulation in this population.

## Data Availability

The datasets used and/or analysed during the current study are available from the corresponding author on reasonable request.

## Abbreviations

AFC: Antral follicle count
AMH: Anti-Müllerian hormone
ASRM: American Society for Reproductive Medicine
BMI: Body mass index
COCs: Cumulus-oocyte complexes
ESHRE: European Society for Human Reproduction and Embryology
FPOS: Follicular-phase ovarian stimulation
FSH: Follicle-stimulating hormone
ICSI: Intracytoplasmic sperm injection
IVF: In vitro fertilization
LH: Luteinizing hormone
LPOS: Luteal-phase ovarian stimulation
MII: Metaphase II
OSI: Ovarian sensitivity index
POR: Poor ovarian response
SART: Society for Assisted Reproductive Technology
SD: Standard deviation
